# Editorial: Biotechnological Applications of Yeasts in Beverages and Food: From Fermentation to Innovation

**DOI:** 10.3389/fmicb.2022.970418

**Published:** 2022-07-04

**Authors:** Lucía M. Mendoza, María Gabriela Merín, Carmela Belloch

**Affiliations:** ^1^Centro de Referencia para Lactobacilos, Consejo Nacional de Investigaciones Científicas y Técnicas (CERELA-CONICET), San Miguel de Tucumán, Argentina; ^2^Facultad de Ciencias Aplicadas a la Industria, Universidad Nacional de Cuyo, San Rafael, Mendoza, Argentina; ^3^Consejo Nacional de Investigaciones Científicas y Técnicas (CONICET), Buenos Aires, Argentina; ^4^Instituto de Agroquímica y Tecnología de Alimentos (IATA-CSIC), Valencia, Spain

**Keywords:** yeasts, starter cultures, non-conventional properties, novel applications, food industry

Fermentation is one of the oldest methods of food preservation used around the world for hundreds of years. The most popular fermentation processes in which yeasts are implied are wine-making and baking. However, these microorganisms are involved in the production of different types of food and beverages such as cheese, dry sausage, beer, cider, among others. In addition, the ability of yeasts, especially *Saccharomyces cerevisiae*, to ferment sugars during the fermentative process is well known. However, in the last years, new properties have been considered in the selection of microorganisms as starter cultures. In this context, the study of characteristics related to the production of antimicrobial peptides and certain aroma compounds, different enzymatic activities even functional properties as probiotics that could have health-promoting effects are being taken into account in the selection of strains for their application in food production.

In many countries, the commercial starter cultures for winemaking are mainly of *S. cerevisiae* being the use and commercialization of other yeast species less extended. For other beverages and fermented foods, the information on yeasts as starter or additive cultures is still scarce. Therefore, the aim of this Research Topic is to highlight the potential applications of different species of yeasts through the current advances in the study of metabolic and functional properties that could increase the knowledge of this research area and promote the use of selected yeasts in the food industry.

Martău et al. proposed to use apple pomace (AP) and wheat flour (WF) to elaborate a novel sourdough by fermentation with *S. cerevisiae* in pure culture and co-culture with lactic acid bacteria (*Fructilactobacillus florum*). AP flour addition had a positive effect on cell viability, mainly in fermentations with 95% WF and 5% AP in co-culture fermentation. The metabolites analysis during fermentation showed that AP may be an important and cheap source of fermentable sugars and organic acids which would increase nutritional sourdough value. In addition, the integration of AP in sourdough fermentation could contribute to manage the apple by-products.

The study by Foster et al. characterized the fermentation performance of traditional Norwegian farmhouse ale yeasts, also known as kveiks, across a wide range of temperature varying from 12 to 42°C. Fermentation kinetics and sugars metabolism together with whole-genome sequences of six kveik strains were recorded and compared to those of four commonly used *S. cerevisiae* ale yeasts. The authors observed strain- and temperature-specific variation in fermentation efficiencies, stress tolerance and cell survival at higher temperatures of the kveik strains. Furthermore, the kveiks displayed an increased level of intracellular trehalose, which could contribute to their thermotolerance. This study showed that kveiks are a suitable option for beer fermentation at high temperatures and can help brewers select optimal temperatures for specific yeast strains to optimize production outcomes.

Catrileo et al. studied the response of *Brettanomyces bruxellensis*, the main spoilage yeast in wine, to the presence of light and p-coumaric acid (pCA) by evaluating the growth and genes expression. When *B. bruxellensis* LAMAP2480 was exposed to two light intensities (2,500 and 4,000 lux) and in the absence of pCA its growth was not detected. In the dark, the addition of pCA (100 mg/L) to the culture medium did not modify the growth kinetics of the strain LAMAP2480. However, in presence of pCA and light the duration of lag phase increased but it could grow to a high concentration of cells. Also, pCA stimulated an antioxidant response with overexpression of the SOD1, GCN4 and ESBP6 genes allowing the growth of *B. bruxellensis*. The yeast response would be related to cross-protection when exposed to both stressors. This study is the first report on the effect of light on *B. bruxellensis* growth as an innovative method to control wine contamination.

Gao et al. investigated the influence of two non-*Saccharomyces* yeasts with high β-glucosidase activity on sensory characteristics of white and red wines. *Meyerozyma guilliermondii* NM218 and *Hanseniaspora uvarum* BF345 improved the floral and fruity aromas of the Cabernet Sauvignon and Chardonnay wines, respectively. Therefore, the fermentation conducted by mixed cultures composed of specific non-*Saccharomyces* yeasts with *S. cerevisiae* is a possible way to improve the aromatic diversity and overall quality of wine products.

Li et al. evaluated the effect of two indigenous strains of *Torulaspora delbrueckii* in sequential fermentation with *S. cerevisiae* on the aromatic profile of Vidal blanc icewine. The authors observed a clear impact of this non-*Saccharomyces* species on the aroma composition of wines, which appeared to be also dependent on the strain. The mixed cultures contributed to a high concentration of some volatile compounds such as 2-phenylethyl alcohol, isoamyl acetate, D-limonene, and cineole. This is the first study on the application of native *T. delbrueckii* in mixed fermentation with *S. cerevisiae* in Vidal blanc icewine, and represents a potential strategy to confer aromatic complexity on icewines and impart their unique regional flavor.

The articles of this Research Topic expand the Frontiers and the knowledge of biotechnological applications of yeasts, confirming the importance of the characterization of these microorganisms involved in traditional fermentations using novel matrix or strategies to improve food and beverages quality. We hope that this Topic will be valuable to the readers of Frontiers in Microbiology and encourages microbiologists and biotechnologists to explore novel applications of yeasts in the food industry.

## Author Contributions

All authors listed have made a substantial, direct, and intellectual contribution to the work and approved it for publication.

## Conflict of Interest

The authors declare that the research was conducted in the absence of any commercial or financial relationships that could be construed as a potential conflict of interest.

## Publisher's Note

All claims expressed in this article are solely those of the authors and do not necessarily represent those of their affiliated organizations, or those of the publisher, the editors and the reviewers. Any product that may be evaluated in this article, or claim that may be made by its manufacturer, is not guaranteed or endorsed by the publisher.

